# An analysis of real-world cost-effectiveness of TAVI in South Africa

**DOI:** 10.5830/CVJA-2013-090

**Published:** 2014-02

**Authors:** Thomas A Mabin, Pascal Candolfi

**Affiliations:** MediClinic Vergelegen, Somerset West, South Africa; Edwards Lifesciences SA, Nyon, Switzerland

**Keywords:** TAVI, cost effectiveness, interventional cardiology, cardiac surgery, aortic stenosis, aortic valve

## Abstract

**Objectives:**

Transcatheter aortic valve implantation (TAVI) has become the standard of care for inoperable patients with severe aortic stenosis and is an alternative to conventional surgery for high-risk aortic valve replacement (AVR) patients. There is a positive correlation between severity of pre-operative patients and hospital costs. The aim of this study was to compare empirically derived costs of the two therapies in South Africa.

**Methods:**

The cost-comparison analysis was performed with a MediClinic database including 239 conventional isolated AVR (cAVR) and 75 TAVI cases. All costs are given in 2011 ZAR. The subset of cAVR patients were derived from the relevant and available information in the database and their costs were compared with TAVI costs.

**Results:**

From the 75 available subjects, mean TAVI costs were ZAR 335.5k ± 47.9k, (median ZAR 326.5k) with a mean (median) ICU and hospital length of stay (LoS) of 2.7 (2.0) and 7.6 (6.5) days, respectively. The mean cAVR cost was lower at ZAR 213.9 ± 87.5k (median ZAR 193.6k) but this included the entire population costs (i.e. low to high surgical risk). When estimating cAVR costs, defined by LoS of more than six and 13 days in the ICU and hospital, respectively, and being over 75 years of age, the estimate increased to ZAR 337.9k, which was above the TAVI mean costs. In-hospital mortality was 5.3 and 7.9% for TAVI and the entire cAVR group, respectively. When considering the subset of cAVR patients most likely to be high risk, it increased to 21.4%.

**Conclusions:**

Within the context of limited clinical data we performed the first attempt at cost-effective analysis of TAVI vs cAVR in South Africa. Treatment of aortic stenosis with cAVR in a *post hoc* defined high-risk patient segment was more expensive than TAVI in South African centres. Despite common perceptions on costs, adoption of TAVI as an alternative, less-invasive therapy that has been clinically proven and recommended by an FDA advisory panel (Partner A) to be at least as effective as cAVR, has a viable economic argument in appropriate patients.

## Abstract

Surgical replacement of defective aortic valves has become almost commonplace in recent years with good outcomes expected.^1^-^3^ A substantial number of patients suffering from severe aortic stenosis are considered inoperable due to existing co-morbidities not allowing a conventional surgical aortic valve replacement (cAVR) intervention. In the latest Euro Heart survey, the estimated prevalence of inoperable patients with severe aortic stenosis was 31.8%.^4^

The Partner Cohort B trial^5^,^6^ randomly assigned patients considered unsuitable candidates for surgery into two groups: standard therapy (including balloon aortic valvuloplasty) or a transcatheter aortic valve implantation (TAVI) via the transfemoral approach. The difference in rate of death from any cause was considerable, with an absolute 20 and 24.7% difference favouring TAVI at one and two years, respectively. TAVI has subsequently emerged as a new standard of care for these patients and is considered one of the most innovative breakthroughs in medicine in recent years.

The Partner Cohort A trial^7^,^8^ randomly assigned high-risk patients and aimed to compare conventional surgery with TAVI (via a transfemoral or transapical approach). Non-inferiority was met and TAVI showed similar clinical benefit – absolute reduction of death from any cause of 2.5% (*p* = 0.45) and 1.1% (*p* = 0.78) at one and two years, respectively. The clinical trade off appeared to be between major vascular complications (more frequent with TAVI) and major bleeding (more frequent surgically). Myocardial infarction at two years, haemodynamics (mean gradient and EOA), anaesthesia and procedure time, recovery (assessed by ICU and hospital length of stay: LoS) were secondary endpoints that also improved with TAVI.

Only limited cost-effectiveness studies with TAVI have been published so far. Reynolds *et al.*^9^ and Watt *et al.*^10^ looked at the cost-effectiveness of TAVI versus medical management for patients ineligible for cAVR, based on the Partner Cohort B trial, from the perspective of the US and UK environments, respectively. The incremental cost-effectiveness ratio (ICER) for TAVI in the US study was estimated at $50 200 per year of life gained or $61 889 per quality-adjusted life years (QALY) gained, and in the UK study at £16 100 in the base case. Both were well within the acceptable threshold.

Gada *et al.*^11^ used a Markov model, also based on the Partner trial and derived the outcomes and costs from 10 000 simulations. They found TAVI and cAVR cost effective when compared with medical management, with incremental cost-effectiveness ratios (ICERs) of $39 964/QALYs and $39 280/QALYs, respectively. TAVI was associated with a QALY gain of 0.06 compared with cAVR but with a greater cost ($59 503 vs $56 339), yielding an ICER of $52 773/QALYs.

We attempted to assess a cost-effective analysis of TAVI versus cAVR in South Africa. TAVI has not yet been fully embraced in the South African market, largely because of concerns on the initial cost of the device, without considering the potential cost savings that may be realised by lowered complication rates and length of hospital stay. The available MediClinic administrative database has limited clinical outcomes but sufficient information to allow for the identification of high-risk cAVR patients who potentially could have been TAVI candidates, and to compare data with the patients who did undergo TAVI.

TAVI has been introduced into clinical practice and is reimbursed in many European countries since its commercial availability in 2007. In the US, FDA approval arrived in November 2011 for inoperable patients and was overwhelmingly (11-0) recommended by an FDA advisory panel in June 2012 for patients at high risk for conventional surgery. The aim of this study was to compare outcomes between TAVI and cAVR in South Africa in order to evaluate the costs and benefits of both treatment options.

## Methods

An initial dataset was obtained from MediClinic, one of the largest South African private hospital groups, and contained billing records on 394 patients who had undergone conventional aortic valve replacement (cAVR) during the period 2009 to 2011 at eight cardiac hospitals, MediClinics Bloemfontein, Heart Hospital, Morningside, Nelspruit, Panorama, Vereeniging, Verglegen and Witwatersrand University Donald Gordon Medical Centre.

From procedural coding we were able to exclude all patients who had undergone concomitant procedures with cAVR (e.g. CABG, ascending aorta) in order to compare more appropriately with TAVI patients who would not electively undergo these additional procedures. This produced a final dataset of 239 isolated cAVR patients. Over the same period the records of 75 TAVI patients were also available.

The dataset included the total costs per patient to the healthcare provider (insurer) without professional fees and no breakdown of cost components was available. Professional fees vary from centre to centre and in order to avoid adding uncertainty to the analyses by including estimates, these were not included. Overall, costs were standardised to 2011 South African Rand (ZAR) using the South African consumer price index (CPI) published on the governmental statistics department website.^12^ For ease of interpretation and at the time of writing, 10 ZAR was approximately equal to € 1 or US$ 1.25.

The database did not provide clinical risk scores, so to compare cAVR with TAVI we excluded the results of those patients who would not be considered for TAVI. Age ≥ 75 years provided the single predictive variable and we used ICU and hospital LoS as surrogates or proxies for indicators of ‘high-risk’ patients.

## Statistical analysis

Quantitative continuous variables are described with means ± standard deviation, and quantitative discrete variables with absolutes and relatives frequencies. Inference statistics comparing continuous variables were made using the *t*-test or Wilcoxon rank sum test as appropriate. To compare discrete variables, Pearson’s chi-squared test with Yate’s continuity correction or Fisher’s Exact test (when count data ≤ 5) were applied. Two-sided tests were used and a type I error significance level of 0.05 was considered. Distributions of quantitative continuous variables are presented graphically with normalised histograms; the *y*-axis is given with densities, ensuring the total area equals one.

When representing the distributions between groups, box-andwhisker plots (boxplots) were chosen. Relationship and linear correlation between quantitative continuous variables were populated and tested with Pearson’s product moment correlation coefficient. Linear models were fitted by ordinary least-square regression estimates and by robust regression using an M estimator (package MASS).^13^ Coefficient estimates are given with standard errors. All analyses were performed with the use of R software, version 2.13.1.^14^

## Results

We obtained a total sample of 75 TAVI and 239 isolated cAVR patients from the period 2009–2011. Descriptive and inference statistics for both TAVI and cAVR groups are presented in Table 1. The mean age for each group was 79.4 ± 7.3 versus 62.3 ± 15.2 years for TAVI and cAVR, respectively. This difference was highly statistically significant (*p* < 0.001). Male gender was more frequent in the cAVR group (59.8%) but less in the TAVI group (44.0%) and this difference was also statistically significant (*p* = 0.023).

**Table 1:** Descriptive table for tavi and cavr groups

*Variable*	*TAVI (n = 75)*	*cAVR (n = 239)*	p*-value*
Age (years)	79.4 ± 7.3	62.3 ± 15.2	< 0.001
Male sex, *n* (%)	33 (44.0)	143 (59.8)	0.023
ICU LoS (days)	2.7 ± 2.8	5.1 ± 6.1	< 0.001
Hospital LoS (days)	7.6 ± 4.9	13.6 ± 9.2	< 0.001
Total costs (ZAR)	335.5k ± 47.9k	213.9k ± 87.4k	< 0.001
In-hospital mortality, *n* (%)	4 (5.3)	19 (7.9)	0.613

Due to limitations in the available clinical data, we were unable to make direct comparisons between groups and it is impossible to draw strong inferences. Surprisingly, in-hospital mortality rates were numerically higher for cAVR than TAVI. Out of the 75 TAVI patients, four (5.3%) died before discharge, compared to 19 (7.9%) for cAVR, although this difference was not statistically significant (*p* = 0.613).

Much less surprisingly, TAVI, a less-invasive procedure, clearly demonstrated faster post-operative recovery. Indeed, the ICU LoS and the hospital LoS were reduced on average by 47.1% (2.7 ± 2.8 vs 5.1 ± 6.1 days, *p* < 0.001) and 44.1% (7.6 ± 4.9 vs 13.6 ± 9.2 days, *p* < 0.001), respectively. These reductions were also robust and were not impacted on by outliers; indeed, when considering the median ICU and hospital LoS, the corresponding reductions were 59.1% (2.0 vs 3.5) and 40.9% (6.5 vs 11.0), respectively. From an absolute perspective, the number of ICU days saved per patient with TAVI was between 1.5 (from medians) and 2.4 (from sample means). Similarly, we could expect a reduction in per-patient hospital LoS of between 4.5 (from medians) and six days (from sample means).

**Fig. 1.**
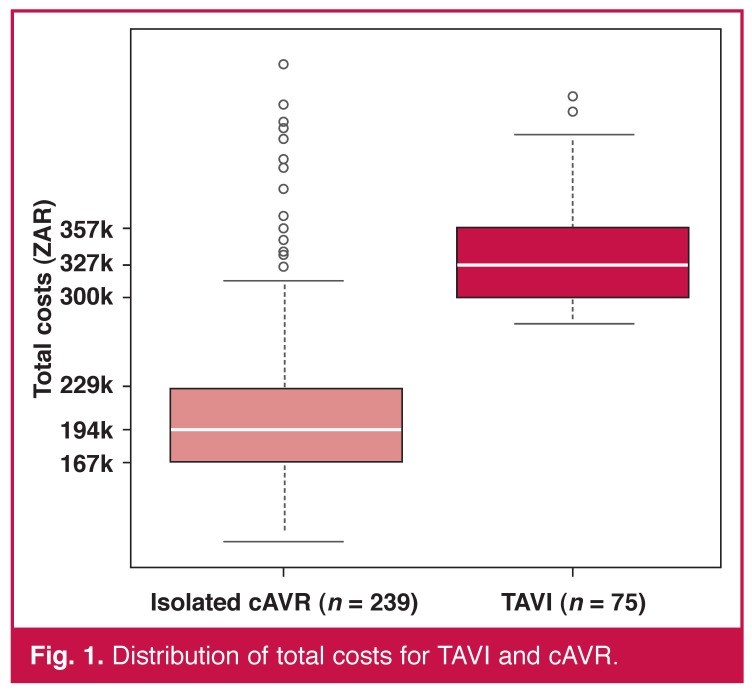
Distribution of total costs for TAVI and cAVR.

The overall average cost per patient to the healthcare provider was ZAR 335.5k ± 47.9k for TAVI and ZAR 213.9k ± 87.4k for cAVR (*p* < 0.001). As we can see from Fig. 1, the cAVR distribution is highly skewed, indicating a presumably heterogeneous population with a wide range of what may be low- to high-risk patients (certainly heterogeneous post-operative outcomes). This seems not to be the case in the TAVI group where the distribution is more symmetrical with fewer outliers.

It seems safe to assume that these groups were not comparable in term of pre-operative severity or risk factors (the average age confirms this to an extent). This is also likely to be the case as TAVI is typically indicated only for inoperable and so-called ‘high-risk’ patients diagnosed with symptomatic severe aortic stenosis. However, cAVR is typically only avoided in patients where the operative risk is considered too high in comparison with the benefits gained, or in those patients determined as inoperable for anatomical reasons.

**Fig. 2.**
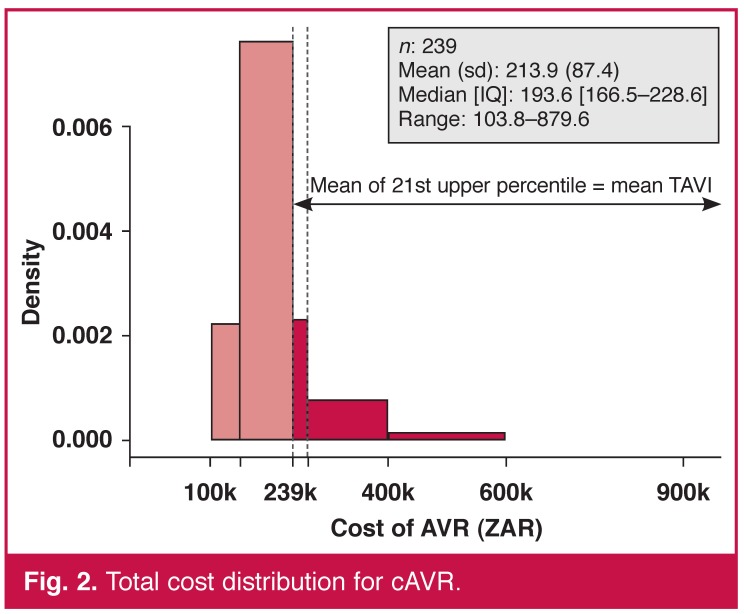
Total cost distribution for cAVR.

Comparing the cost distributions, we see that the upper 21st percentile of the cAVR sample equals the same average costs as the entire TAVI group, that is, the most costly 50 patients undergoing cAVR generated the same average costs as the 75 TAVI patients (Fig. 2). We could then state that on average, one patient out of five could be treated with a less-invasive therapy associated with lower in-hospital mortality and faster recovery at an equal cost.

**Fig. 3.**
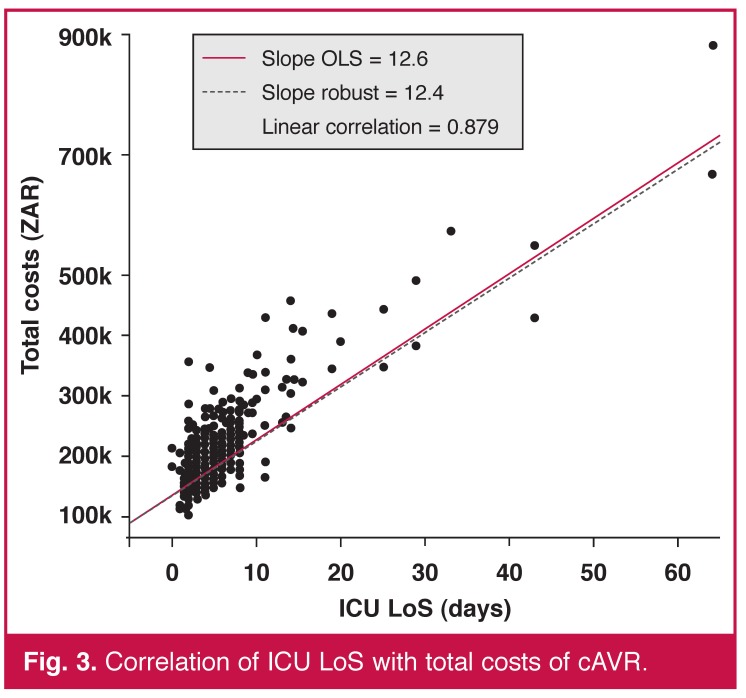
Correlation of ICU LoS with total costs of cAVR.

**Fig. 4.**
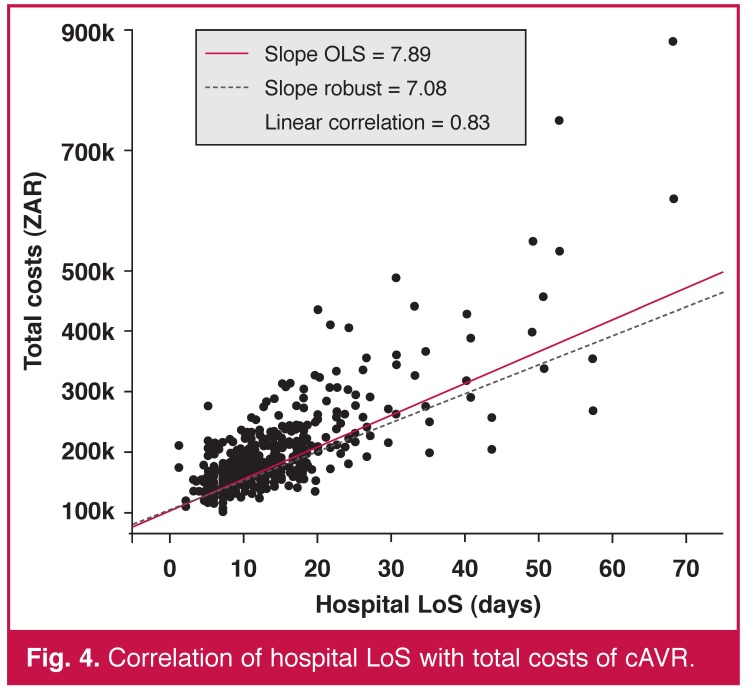
Correlation of hospital LoS with total costs of cAVR.

In our sample, ICU and hospital LoS were also heavily positively correlated with cost and highly statistically significant in both cases (*p* < 0.001). Figs 3 and 4 illustrate the linear relationship with linear regression models fitted. Each additional ICU and hospital day increased the total costs by ZAR 12.6k (0.44) and ZAR 7.9k (0.34), respectively.

**Fig. 5.**
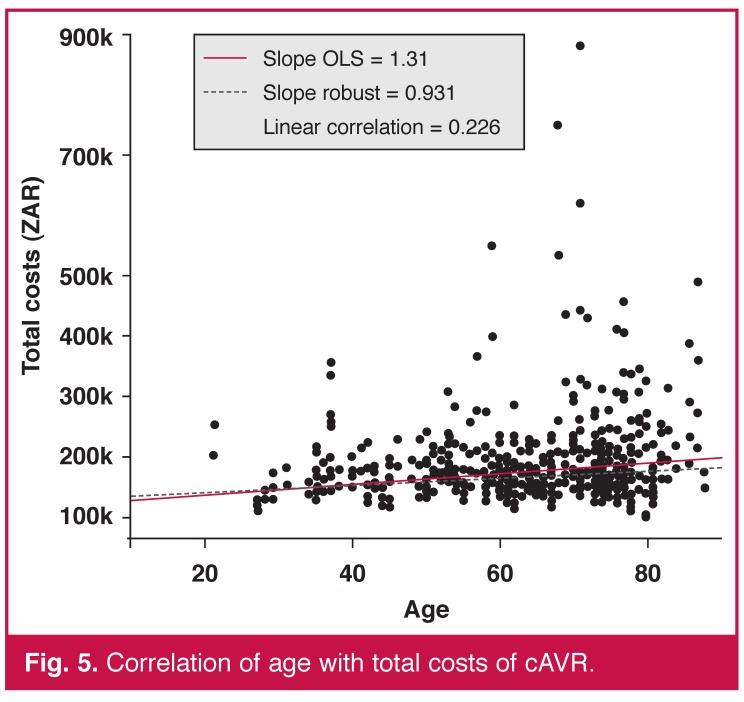
Correlation of age with total costs of cAVR.

**Fig. 6.**
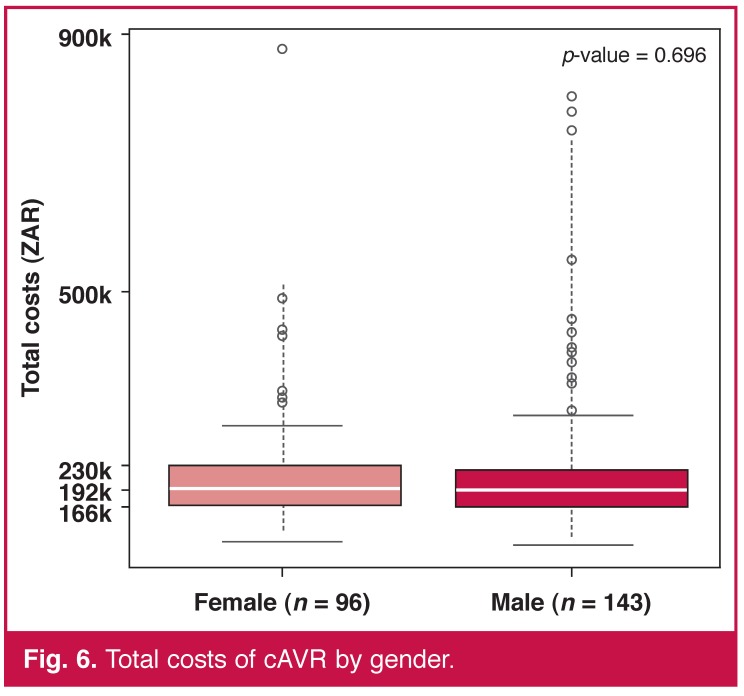
Total costs of cAVR by gender.

By contrast, age and gender, our only two pre-operative variables available, were not predictors of cost. The linear correlation between age and total costs was statistically significant (*p* < 0.001) but the coefficient, also positive, was low in comparison with the previous one of 0.226 (Fig. 5). The total cost distributions were similar for both sexes (Fig. 6).

## Stratifying patient groups

In order to make a comparison of costs between procedures, we attempted to use the available data to select those cAVR patients most likely to have also been eligible for a TAVI (assuming typical selection criteria). Although we had very few predictive risk factors, we examined the literature for data on LoS to use as proxies for defining a more ‘high-risk’ subgroup of cAVR patients.

As a baseline for LoS with both TAVI and cAVR we have data from cohort A of the Partner trial,^7^,^8^,^15^ which gives values for ICU and hospital LoS. The means (medians) for the high-risk cAVR patients were 8.4 (5.0) ICU days and 16.7 (12.0) hospital days. Additionally, Thourani^16^ described the outcomes in surgical cohorts undergoing AVR who would meet the qualifications for transcatheter valve therapies and was able to retrieve data on 159 patients from January 2002 to December 2007 at four US academic institutions. Here the mean ICU and hospital LoS were 6.9 ± 10.6 and 12.6 ± 11.1 days, respectively; very similar to the Partner trial results. In a study from Switzerland, Wenaweser^17^ determined that a group of cAVR patients, who were younger and had lower predicted peri-operative risks (logistic EuroSCORE 12.5 ± 8.2%) compared with two other groups (TAVI or medical management), had a mean hospital LoS of 15.0 ± 20.2 days.

The French Ministry of Health (MoH) records all procedures in the administrative PMSI database. The database is mandatory for each centre in order to be reimbursed. With the corresponding DRGs and specific procedure codes, we were able to select and retreive basic information from all isolated cardiac surgery procedures in France in 2010. This analysis gave us a real-life picture of the French cardiac surgery environment. Clinical data were limited, as was to be expected, but in-hospital mortality, hospital LoS and procedure costs were available.

Specific risk scores, such as the logistic EuroSCORE or the STS score were not available, but we were able to populate the Charlson score, an administrative score calculated from the ICD codes. This score has been validated in different publications 18,19 and is used as a risk factor in various areas such as oncology,^20^ acute myocardial infarction,^21^ ischaemic stroke^22^ and infection.^23^ By using the score we gained an idea of the average pre-operative risk score for each of the four severity levels derived post surgery, leading to a specific reimbursement DRG. In the France 2 TAVI registry, including all TAVI procedures between 2010 and 2011,^24^ the Charlson score was calculated for 2 568 patients (unpublished data) in an intermediate report sent to the HAS (Haute Autorité de Santé or French National Authority for Health) to evaluate the technology, and the sample mean was 2.6 ± 2.2 (median = 2.0).

Table 2 illustrates the strong correlation between the Charlson score, in-hospital mortality, hospital LoS, procedure costs and severity level defined postoperatively. From this table, it seems reasonable to assume that the majority of TAVI candidates would have been severity level 3 and 4. Therefore we could assume that around 15% of the entire cAVR population could have been considered high risk and would fit the TAVI indications. For these patients the ‘real-life’ clinical outcomes were far from what can be found in the literature, with average hospital LoS above 20 days and the in-hospital mortality rate up to 20%. Surprisingly, age did not seem to have a strong impact on hospital LoS, in-hospital mortality rate or procedure costs.

**Table 2:** Cardiac surgery (cavr) from the French Moh database

*Severity*	*Patients*		*Age (years)*	*Charlson*	*Hospital mortality (%)*	*Hospital LoS (days)*	*Total costs (ZAR)*
	*n*	*%*					
Level 1	2 537	20.3	69.2	1.02	1.66	10.7	14 365
Level 2	5 805	46.4	73.0	1.74	2.48	12.5	16 304
Level 3	2 733	21.8	73.6	2.47	8.89	16.8	22 196
Level 4	1 437	11.5	71.9	2.84	21.85	27.9	32 250
Total	12 512	100.0	72.3	1.88	5.94	14.8	19 029

To produce the most conservative estimate of the cAVR group comparable with TAVI, if we take our sample with the proxy thresholds defined above, i.e. ICU and hospital LoS above six and 13 days, respectively, patients ≥ 75 years, we derive our subset of high-risk patients. Table 3 shows the number of patients, in-hospital mortality rate and average costs for each criterion considered.

**Table 3:** Total cost and in-hospital mortality rate for our three proxies

*Proxy*	*Patients*		*Hospital mortality (%)*	*Total costs (ZAR)*
	*n*	*%*		
ICU LoS > 6 days	47	19.7	19.1	320.2k ± 136.9k
Hospital LoS > 13 days	85	35.6	9.4	276.9k ± 116.3k
Age > 75 years	49	20.5	10.2	236.2k ± 88.7k
All proxies combined	14	5.9	21.4	337.9k ± 80.9k

When combining all three parameters, 14 patients (5.9%) were found, which is not surprising, as we could expect that some of these patients were treated with TAVI. The average costs estimate for our high-risk patients was ZAR 337.9k ± 80.9k, marginally higher than the average TAVI costs (ZAR 335.5k). However, very strikingly, the clinical outcome was much worse, with an in-hospital mortality rate of 21.4%, more than four times that of TAVI [RR = 0.25 (0.06–0.99), *p* = 0.075].

Overall, with limited available data, and by using proxies to derive a subset of high-risk patients, we concluded that TAVI is likely to be cost-effective versus cAVR. Using the most conservative estimates, we predict a small number of patients who could benefit from TAVI versus cAVR, with a lower mortality rate at, on average, lower costs.

## Discussion

In our sample of TAVI and cAVR hospital records from an administrative database, we found that TAVI patients were on average older, had reduced ICU and hospital LoS, non-statistically significantly reduced mortality rates and higher costs than cAVR patients. However, by trying to identify those cAVR subjects who would most likely have been candidates for TAVI, we projected that a small group would have clinically benefitted from TAVI (lower mortality rate) at a reduced cost to the funder.

The sample’s mean TAVI costs were higher than those of cAVR, but the latter group was more heterogeneous and presumably included a wide range of patients in terms of pre-operative severity levels, which means a direct comparison between the two groups is difficult. In-hospital mortality rate was lower in the TAVI group, which was not expected, as cAVR has a much broader utility and would be expected to be used in lower-risk patients.

The average of the upper 21st percentile cAVR cost distribution was equal to the average TAVI cost, so from a purely economic perspective, we could assign one patient out of five with a novel, less-invasive treatment, ensuring faster recovery with a reduction of over 40% in ICU and hospital LoS at no additional cost. This substantial reduction was expected despite the difference in age between the two groups and reflects the more rapid and typically less-invasive nature of the procedure. It is also supported by numerous clinical data, although it may be confounded by local guidelines and operational practice, depending on the vagaries of the healthcare system (e.g. reimbursement practices, discharge to alternative local facilities).

When estimating the high-risk cAVR costs, defined by patients staying more than six and 13 days in ICU and hospital, respectively (as was found in the Partner Cohort A trial), and being over 75 years of age, the estimate increased just above the TAVI mean cost, and the in-hospital mortality rate increased to 21.4%, a four-fold increase compared with the TAVI group, and a rate similar to that of the French MoH level 4 severity group.

Our findings are not dissimilar to others in the literature. For example, Arnaoutakis^25^ studied the relationship between STS score and hospital charges. In their analysis the authors showed that the median hospital charges for patients with risk scores above and below 10% were US$ 88 241 and US$ 42 785, respectively, a relative difference above 100%. In a multivariate regression model they found that each 1% increase in STS risk score was associated with an additional US$ 3 000. Additionally, Toumpoulis^26^ found a significant correlation between additive EuroSCORE and hospital LoS, as to be expected, whereby a higher-risk score leads to higher costs and extended LoS.

There were several limitations with this work, most notably, the restricted clinical data available to us from an administrative database. The task of making any comparison between patients according to typical clinical criteria was therefore more difficult, but also *post hoc* matching of records has its own limitations. Of enormous benefit and in contrast to most economic analyses, we had access to precise billing data. Typically, other analyses use proxy cost data from other studies to estimate the total cost of a procedure, but here we had the total cost from admission to discharge, albeit without details of professional fees. It should be noted that the omission of professional fees favours cAVR, as these fees are likely to be higher than for TAVI. An additional limitation is that we do not have any data on re-admission after discharge. No differences are expected between the groups but additional analyses would help to quantify this important outcome.

The transferability of the conclusions from this study are also limited, in common with those from any economic evaluation. We acquired our data from a South African administrative database and the clinical practice underpinning the costs derived may differ from other settings. However, this also provided a major strength of the study in that the data we had was derived from South African clinical practice and the results were not confounded by the use of unrelated data.

The adoption of any new technology is challenging and especially if it is a ground-breaking intervention that challenges treatment paradigms. However, the data on TAVI from South Africa show that TAVI costs are much more predictable than cAVR, which should greatly aid planning and implementation. At best it would appear that TAVI could reduce costs to funders and improve outcomes in appropriately selected individuals.

## Conclusion

Within the context of limited clinical data, we performed the first attempt at cost-effective analysis of TAVI versus cAVR in South Africa. Treatment of aortic stenosis with cAVR in a *post hoc* defined high-risk patient segment is more expensive than TAVI in South African centres. Despite common perceptions on costs, adoption of TAVI as an alternative, less-invasive therapy that has been proven clinically and recommended by an FDA advisory panel (Partner A) to be at least as effective as cAVR has a viable economic argument in appropriate patients.
